# Oleanolic acid and ursolic acid inhibit peptidoglycan biosynthesis in *Streptococcus mutans* UA159

**DOI:** 10.1590/S1517-838246246220130209

**Published:** 2015-06-01

**Authors:** Soon-Nang Park, Sug-Joon Ahn, Joong-Ki Kook

**Affiliations:** 1 Korean Collection for Oral Microbiology and Department of Oral Biochemistry, School of Dentistry, Chosun University, Gwangju, >Republic of Korea, Korean Collection for Oral Microbiology and Department of Oral Biochemistry, School of Dentistry, Chosun University, Gwangju, Republic of Korea.; 2 Seoul National University, School of Dentistry, Seoul National University, Seoul, >Republic of Korea, Dental Research Institute, School of Dentistry, Seoul National University, Seoul, Republic of Korea.

**Keywords:** ursolic acid, oleanolic acid, peptidoglycan biosynthesis, *Streptococcus mutans* UA159

## Abstract

In this study, we revealed that OA and UA significantly inhibited the expression of most genes related to peptidoglycan biosynthesis in *S. mutans* UA159. To the best of our knowledge, this is the first report to introduce the antimicrobial mechanism of OA and UA against *S. mutans*.

Ursolic acid (UA, (3β)-hydroxy-urs-12-en-28-oic acid), oleanolic acid (OA, 3β-3-hydroxyolean-12-en-28-oic acid), and betulinic acid (BA, 3β-3-hydroxy-lup-20(29)-en-28-oic acid) are derivatives of triterpenoid saponins (Liu, 1995; Fontanay *et al.*, 2008). These compounds are naturally found in a large variety of vegetables, medicinal herbs, and plants that have been investigated for antibacterial activity (Fontanay *et al.*, 2008). The antimicrobial activity of OA and UA is stronger than that of BA (Fontanay *et al.*, 2008). OA and UA inhibit growth of Gram-positive bacteria but not Gram-negative bacteria (Fontanay *et al.*, 2008), but their antimicrobial mechanism is unknown. Previously, we reported that OA and UA have strong antimicrobial activity against *Streptococcus mutans* (Kim *et al.*, 2010; Kim *et al.*, 2011) but BA had no antimicrobial activity against *S. mutans* (data not shown). One of the characteristic differences between Gram-positive and Gram-negative bacteria is the thickness of the peptidoglycan layer. Considering this difference, it has been suggested that the mechanism of OA and UA antimicrobial activity may be related to inhibition of peptidoglycan biosynthesis (Horiuchi *et al.*, 2007). Thus, the objective of this study was to investigate the effect of BA, OA, and UA on peptidoglycan biosynthesis in *S. mutans* UA 159 using the quantitative real-time polymerase chain reaction (qPCR) method to identify the antimicrobial mechanism against *S. mutans*.

*S. mutans* UA159 was a kind gift from Dr. Robert A. Burne, Department of Oral Biology, College of Dentistry, University of Florida. The strain was cultured in brain heart infusion (BHI) broth (Difco, Lab., Detroit, MI, USA) or on BHI agar plates in a 37 °C incubator.

Overnight cultures (1 mL) of *S. mutans* UA 159 were transferred to 9 mL BHI broth and grown at 37 °C to the mid-log phase (OD_600_ = 0.35). UA (Sigma, St. Louis, MO, USA), OA (Sigma), or BA (Sigma) solutions were added to each tube to a final concentration of 64 μg/mL. The bacterial culture solutions were incubated in a 37 °C incubator for 90 min and then harvested by centrifugation at 10,000 × *g* for 10 min at 4 °C. After discarding the supernatant, liquid nitrogen was immediately added to the tube. The frozen bacterial pellets were homogenized using a mortar and pestle (Smile Science, Seoul, Korea). Total RNAs were extracted from the homogenized bacteria following the manufacturer's instructions of the RNAqueous^®^ kit (Ambion, Austin, TX, USA). DNase I treatment was conducted to completely remove the bacterial genomic DNAs using a TURBO DNA-*free*™ Kit (Ambion) according to the manufacturer's protocol. RNA concentration was determined at 260 nm 280 nm with a UV spectrophotometer (Ultrospec 2000, Pharmacia Biotech., UK).

We consulted the KEGG pathway database to determine the target genes related to *S. mutans* UA159 peptidoglycan biosynthesis (KEGG pathway). qPCR primers were designed based on the nucleotide sequences of the target genes (GenBank accession no. AE014133.2) using the MegAlign and PrimerSelect programs (Lasergene™ 8.0, DNAStar, Inc., Madison, WI, USA) (Table 1). The qPCR primers were synthesized by Bioneer Co. (Daejeon, Korea).

**Table 1:** Quantitative real-time polymerase chain reaction (qPCR) primers used in this study.

Genes	Primer names and oligonucleotide sequences (5 – > 3)	Size of amplicon (bp)
*glmU*	UA159-glmU-F: TGATCCTTTCGGTTATGGTCGTAT UA159-glmU-R: CGTTCCCGTGTTAATTTCTTTGA	118
*murA*	UA159-murA-F1: CCTCCGGGCATAGAAACCTTAG UA159-murA-R1: ATTTTAGATGTGGCTCCTTATGAA	110
*murB*	UA159-murB-F1: CGAGATATGCGCTTTGGTT UA159-murB-R1: AACGCTGCATTTCCTGACTG	121
*murC*	UA159-murC-F1: GGTAACCATTTTCGAGAGCATAGG UA159-murC-R1: ACACGGATTGGAAAAAGCAGGAA	140
*murC2*	UA159-murC2-F: CAAATGGATCGTTACGGTGAAAT UA159-murC2-R: TTGGCGTTAAAGAGTGGGCTATC	116
*murD*	UA159-murD-F: TCAAACGCAGGCAAAGATTATTC UA159-murD-R: TTTCAACATTATGGCTTCCTGGTA	143
*murE*	UA159-murE-F1: CGTCCGGCCATGATTTCTACCA UA159-murE-R1: GCTCTCGGGGGTTGTTAGTTTTGA	81
*alr*	UA159-alr-F: AGAAGGCGGGAGCGACTG UA159-alr-R: CAATTGACTGATAAGGGTGGTAAA	116
*ddI*	UA159- D-Ala-F: AGATGTGGCTTTTTATGATTACGA UA159- D-Ala-R: AAAGTCCACAGCAGCCCAAAGT	146
*murI*	UA159-murI-F: AATGGGCCGTAAAAGTGGATAATG UA159-murI-R: TGCCCCAAATTTGTTCCTAT	147
*murF*	UA159-murF-F1: GGTGCTGGCAGATATGAAAGAAT UA159-murF-R1: AGTGGTCCAAAAAGAAAAATACGA	111
*bacA*	UA159-bacA-F: AACGGCCTTTTTCATTGGTCTG UA159-bacA-R: GTTGCGACTTGCCGACTGG	114
*mraY*	UA159-mraY-F: CTTTGCATTTGGGCGTGTTTTA UA159-mraY-R: GAAGCCAAGCCATCAATACCATC	100
*murG*	UA159-murG-F: AAGGTGGGTTTGTTTCAGTTCC UA159-murG-R: GCAATTCGGTTAGCCAGTCC	106
*murM*	SM159-murM-F: TATTTTTGGCAGTACGGATGGAT SM159-murM-R: ATGGCTGGGACAAACAAGTGA	131
*murN*	UA159-murN-F: AAACAAAGAGACTGCCTGCTAAGA UA159-murN-R: AAAACCAATTGCGTGAACTGTC	123
*pbp1a*	UA159-pbp1a-F: TTGATGCCGCTGGATTAGATACTG UA159-pbp1a-R: CACTGCTGGCACCATACTTTTGTT	132
*pbp1b*	UA159-pbp1b-F: ATGGTGCTTCTCTTGATGATG UA159-pbp1b-R: CAAAGGCGTGATTATTCTGAT	132
*pbp2a*	UA159-pbp2a-F: TATGTTGAAGGGTCCGGGTATCTA UA159-pbp2a-R: CGGTTCCCCATTCCCACTTT	150
*pbp2b*	UA159-pbp2b-F1: ACCGCGTTGGAACATCTTATCT UA159-pbp2b-R1: TAGTCCCTTTGGCAGTGGTCTTA	129
*pbp2X*	UA159-pbp2X-F: AAAGACGGACAAGTGACCTACCAA UA159-pbp2X-R: CCATCTGCGTTTCCAAATAAGTCT	145
*dacA*	UA159-dacA-F: GTACCGCCAACTTTTTCAGC UA159-dacA-R: CAGCGCCAGCAATGTCC	120
16S rDNA	SM159-16S -F: CACACCGCCCGTCACACCAC SM159-16S -R: CCAGCCGCACCTTCCGATACG	137

*glmU*, UDP-*N*-acetylglucosamine pyrophosphorylase gene; *murA*, UDP-*N*-acetylglucosamine 1-carboxyvinyltransferase gene; *murB*, UDP-*N*-acetylenolpyruvoylglucosamine reductase gene; *murC*, UDP-*N*-acetyl muramate-alanine ligase gene; *murC2*, UDP-*N*-acetylmuramyl tripeptide synthetase gene; *murD*, UDP-*N*-acetylmuramoyl-L-alanyl-D-glutamate synthetase gene; *murE*, UDP-*N*-acetylmuramoyl-L-alanyl-D-glutamate-L-lysine ligase gene; *alr*, alanine racemase gene; *ddI*, D-alanine-D-alanine ligase gene; *murI*, glutamate racemase gene; *murF*, UDP-*N*-acetylmuramoyl-tripeptide—D-alanyl-D-alanine ligase gene; *bacA*, undecaprenyl pyrophosphate phosphatase gene; *mraY*, phospho-*N*-acetylmuramoyl-pentapeptide-transferase gene; *murG*, undecaprenyldiphospho-muramoylpentapeptide beta-*N*-acetylglucosaminyltransferase gene; *murM*, UDP-*N*-acetylmuramoylpentapeptide-lysine N6-alanyltransferase gene; *murN*, alanine adding enzyme gene; *pbp1a*, penicillin binding protein 1a gene; *pbp1b*, penicillin binding protein 1b gene; *pbp2a*, penicillin binding protein 2a gene; *pbp2b*, penicillin binding protein 2b gene; *pbp2X*, penicillin binding protein 2× gene; *dacA*, serine-type D-Ala-D-Ala carboxypeptidase (DD-transpeptidase, DacA) gene; 16S rDNA, 16S ribosomal RNA gene.

First-strand cDNAs synthesis was performed with 4 μg of total RNA, random hexamers, and an *AccuPower* ^®^ *RocketScript*™ RT PreMix (Bioneer Co., Deajeon, Korea) using the *Exicycler*™ 96 Real-Time Quantitative Thermal Block (Bioneer Co.) and following the manufacturer's protocol. qPCR was performed with the AccuPower^®^ *GreenStar*™ qPCR PreMix (Bioneer Co.) using the *Exicycler*™ 96 Real-Time Quantitative Thermal Block. Each PCR reaction was performed in a total volume of 20 μL containing 1 μL each of the forward and reverse primers (final concentration, 500 nM each), 0.63 μL cDNA, and the appropriate dose of sterilized DNase-RNase-free water in PreMix PCR tubes. The qPCR conditions were initial denaturation at 95 °C for 3 min, 40 cycles of denaturation at 95 °C for 15 sec, primer annealing and extension at 55 °C for 15 sec, and final cooling at 25 °C for 1 min. Each qPCR reaction was performed in triplicate.

The expression levels of each target gene in the group were normalized with those of 16S rDNA. The normalized expression of each gene (N) was determined as:

$$\text{N}=2^{\Delta }\text{Ct}=2^{\left(\text{Ct value of the target gene - Ct value of }16\text{S rDNA}\right)}$$

Relative expression of the target genes between each experimental group (BA, OA, or UA) and the negative control group (1% DMSO) was calculated as [N of each experimental group (BA, OA, or UA)/N of the negative control group].

Data are expressed as mean ± standard deviation. Independent sample two-tailed *t*-tests were conducted to calculate the *p*-values using SPSS version 12 software (SPSS Inc., USA). A p-value ≤ 0.05 was considered statistically significant.

We determined the BA, OA, and UA concentration of 64 μg/mL by investigating the antimicrobial mechanism against *S. mutans* UA159 by calculating the growth curve at 16, 32, and 64 μg/mL BA, OA, and UA for 60, 90, 120, and 180 min in *S. mutans* UA159 at the mid-log phase (OD_600_ = 0.35). The growth of *S. mutans* UA159 was in a static (inhibited) state at concentrations of 64 and 32 μg/mL of OA and UA, but in the exponential state at concentrations of 64 and 32 μg/mL in the BA-treated and control groups (data not shown). We decided to further explore using 64 μg/mL BA, OA, and UA.

The synthesis of UDP-*N*-acetylglucosamine (UDP-GlcNAc) and UDP-*N*-actetylmuramic acid (UDP-MurNAc), the building blocks of peptidoglycan, is the first phase of first stage (stage I) of intracellular peptidoglycan assembly (Bugg and Walsh, 1992). UDP-GlcNAc is synthesized from GlcNAc-1-P by UDP-*N*-acetylglucosamine pyrophosphorylase (GlmU). UDP-MurNAc is synthesized from UDP-GlcNAc by UDP-*N*-acetylglucosamine 1-carboxyvinyltransferase (MurA) and UDP-*N*-acetylenolpyruvoylglucosamine reductase (MurB) (Bugg and Walsh, 1992). The data showed that UA and OA, but not BA, significantly downregulated *glmU*, *murA*, and *murB* expression (Figure 1A). Interestingly, *glmU* was overexpressed by a factor of two in the BA-treated group compared to that in the control group. The second phase of stage I of peptidoglycan biosynthesis is the assembly of the UDP-MurNAc-pentapeptide (Bugg and Walsh, 1992). The D-amino acids, D-alanine, D-glutamate, and DL-diaminopimelate, occur in peptidoglycan (Bugg and Walsh, 1992). For *S. mutans* UA159, D-alanine and D-glutamate are catalyzed by alanine racemase (Alr) and glutamate racemase (MurI), respectively (the KEEG pathway). Biosynthesis of DL-diaminopimelate (DL-DAP) is catalyzed by DAP epimerase or meso-DAP D-dehydrogenase (Bugg and Walsh, 1992). These genes are missing in *S. mutans* UA159, even though UDP-*N*-acetylmuramyl tripeptide synthetase (MurC2) which catalyzes adding DL-DAP to UDP-MurNAc-L-alanine-D-glutamate occurs (KEEG Pathway). The reason for the discrepancy is unclear, but it is possible that DL-DAP is synthesized through an unknown pathway or that DL-DAP is not used in peptidoglycan synthesis in *S. mutans* UA159. Generally, DL-DAP is added as the third residue in the pentapeptide of Gram-negative bacteria and some Gram-positive bacteria, whereas lysine is added in other Gram-positive bacteria (http://www.enzyme-database.org/reaction/polysacc/PepGly1.html). Using these D-amino acids and D-Ala-D-Ala formed by D-alanine-D-alanine ligase (DdI), two types of UDP-MurNAc-pentapeptide, UDP-MurNAc-L-Ala-γ-D-Glu-DL-DAP-D-Ala-D-Ala (UDP-MurNAc-pentapeptide I) and UDP-MurNAc-L-Ala-γ-D-Glu-DL-DAP-D-Ala-D-Ala (UDP-MurNAc-pentapeptide II) are synthesized by UDP-*N*-acetyl muramate-alanine ligase (MurC), UDP-*N*-acetylmuramoyl-L-alanyl-D-glutamate synthetase (MurD), MurC2 or UDP-*N*-acetylmuramoyl-L-alanyl-D-glutamate-L-lysine ligase (MurE), and UDP-*N*-acetylmuramoyl-tripeptide-D-alanyl-D-alanine ligase (MurF) (the KEGG Pathway). The data showed that second stage-related genes were downregulated by OA and UA, but *murE* was downregulated only by BA (Figure 1A). These results reveal that OA and UA inhibited expression from the first stages of *S. mutans* UA159 peptidoglycan biosynthesis, whereas BA induced overexpression of UDP-GlcNAc, the first substrate for peptidoglycan synthesis.

**Figure 1:**
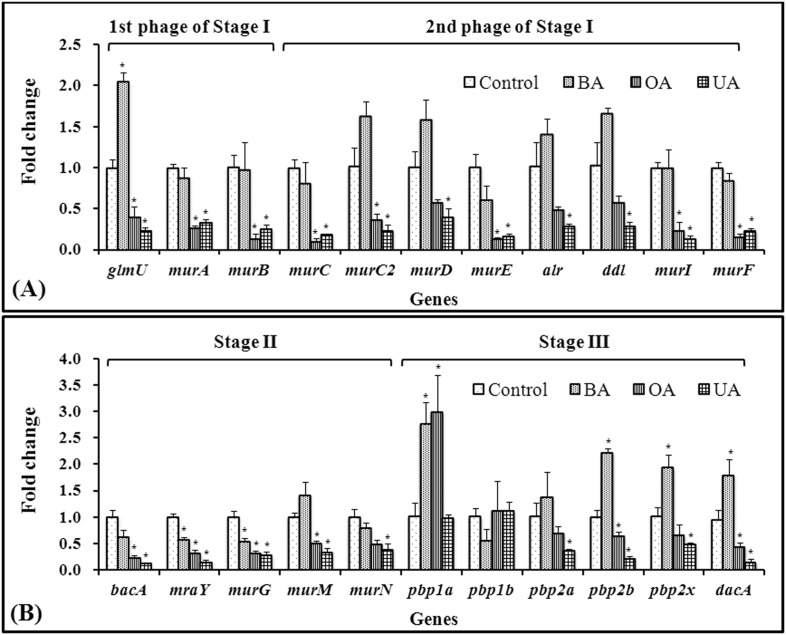
Relative expression of genes related to peptidoglycan biosynthesis, (A) stage I and (B) stage II and III, in *Streptococcus mutans* UA159 following treatment with betulinic acid (BA), oleanolic acid (OA), or ursolic acid (UA). *glmU*, UDP-N-acetylglucosamine pyrophosphorylase; *murA*, UDP-N-acetylglucosamine 1-carboxyvinyltransferase; *murB*, UDP-N-acetylenolpyruvoylglucosamine reductase; *murC*, UDP-N-acetyl muramate-alanine ligase; *murC2*, UDP-N-acetylmuramyl tripeptide synthetase; *murD*, UDP-N-acetylmuramoyl-L-alanyl-D-glutamate synthetase; *murE*, UDP-N-acetylmuramoyl-L-alanyl-D-glutamate-L-lysine ligase; *alr*, alanine racemase; *ddI*, D-alanine-D-alanine ligase; *murI*, glutamate racemase; *murF*, UDP-N-acetylmuramoyl-tripeptide—D-alanyl-D-alanine ligase; *bacA*, undecaprenyl pyrophosphate phosphatase; *mraY*, phospho-N-acetylmuramoyl-pentapeptide-transferase; *murG*, undecaprenyldiphospho-muramoylpentapeptide beta-N-acetylglucosaminyltransferase; *murM*, UDP-N-acetylmuramoylpentapeptide-lysine N6-alanyltransferase; *murN*, alanine adding enzyme; *pbp1a*, penicillin binding protein 1a; *pbp1b*, penicillin binding protein 1b; *pbp2a*, penicillin binding protein 2a; *pbp2b*, penicillin binding protein 2b; *pbp2x*, penicillin binding protein 2x, *dacA*, serine-type D-Ala-D-Ala carboxypeptidase (DD-transpeptidase, DacA). ^*^p ≤ 0.05, significantly downregulated or upregulated compared with the control group.

The second stage (stage II) of peptidoglycan biosynthesis is transport of the MurNAc-pentapeptide portion of the UDP-MurNAc-pentapeptide across the cytoplasmic membrane by a undecaprenyl lipid carrier, undecaprenyl phosphate (UP), which is synthesized from undecaprenyl pyrophosphate (UPP) by undecaprenyl pyrophosphate phosphatase (BacA) (Higashi *et al.*, 1970; Bouhss *et al.*, 2008). The coupling of UDP-MurNAc-pentapeptide to UP to produce UPP-MurNAc-pentapeptide (Lipid I) and UMP is catalyzed by phospho-*N*-acetylmuramoyl-pentapeptide-transferase (MraY). Then, another glucosamine residue, GlcNAc, is attached to Lipid I by undecaprenyldiphospho-muramoylpentapeptide beta-*N*-acetylglucosaminyltransferase (MurG) and forms UPP-MurNAc-(GlcNAc)-pentapeptide (Lipid II). 2 L-Alanine (L-Ala) is added to UDP-MurNAc-(GlcNAc)-L-Ala-γ-D-Glu-L-Lys-D-Ala-D-Ala by UDP-*N*-acetylmuramoylpentapeptide-lysine N6-alanyltransferase (MurM) and another alanine-adding enzyme (MurN) to form UDP-MurNAc-(GlcNAc)-L-Ala-γ-D-Glu-L-Lys-(L-Ala)_2_-D-Ala-D-Ala (the KEGG pathway). The data showed that OA and UA significantly inhibited expression of *bacA*, *mraY*, *murG*, *murM*, and *murN* and that BA also downregulated these genes, except *murM* and *murN* (Figure 1B). These findings reveal that OA, UA, and BA inhibited translocation of the MurNAc-pentapeptide, an intermediate or monomer of peptidoglycan in the cell wall.

The last stage (stage III) of peptidoglycan biosynthesis is transglycosylation, transpeptidation, and trimming of the peptidoglycan, which are catalyzed by penicillin-binding proteins (PBPs) and transpeptidase (Pinho *et al.*, 2001; Lee *et al.*, 2003; Mainardi *et al.*, 2005; Fan *et al.*, 2007). *S. mutans* UA159 has five PBPs such as PBP1a, PBP1b, PBP2a, PBP2b, and PBP2X, as well as serine-type D-Ala-D-Ala carboxypeptidase (DacA), which is involved in the last stage of peptidoglycan biosynthesis (the KEEG pathway). The data showed that these genes, except PBP1a and PBP1b, were downregulated 52–85% and 31–56% in the OA and UA groups compared to those in the control group. Our qPCR data showed that OA and UA significantly downregulated the PBP genes, except *pbp1a* and *pbp1b*, whereas BA significantly upregulated these genes, except *pbp1b* and *pbp2a* (Figure 1B). These results indicate that OA and UA inhibit polymerization between a newly synthesized peptidoglycan monomer and the existing peptidoglycan, and that BA increased polymerization in *S. mutans* UA159.

In summary, OA and UA inhibited the expression of peptidoglycan biosynthesis-related genes of *S. mutans* UA159 at the transcriptional level which might be the one of the antimicrobial mechanism of OA and UA against *S. mutans*.

## References

[B01] Bouhss A, Trunkfield AE, Bugg TD (2008). The biosynthesis of peptidoglycan lipid-linked
intermediates. FEMS Microbiol Rev.

[B02] Bugg TD, Walsh CT (1992). Intracellular steps of bacterial cell wall peptidoglycan
biosynthesis: enzymology, antibiotics, and antibiotic
resistance. Nat Prod Rep.

[B03] Fan X, Liu Y, Smith D (2007). Diversity of penicillin-binding proteins. Resistance factor FmtA
of *Staphylococcus aureus*. J Biol Chem.

[B04] Fontanay S, Grare M, Mayer J (2008). Ursolic, oleanolic and betulinic acids: antibacterial spectra and
selectivity indexes. J Ethnopharmacol.

[B05] Higashi Y, Strominger JL, Sweeley CC (1970). Biosynthesis of the peptidoglycan of bacterial cell walls. XXI.
Isolation of free C55-isoprenoid alcohol and of lipid intermediates in
peptidoglycan synthesis from *Staphylococcus
aureus*. J Biol Chem.

[B06] Horiuchi K, Shiota S, Hatano T (2007). Antimicrobial activity of oleanolic acid from Salvia officinalis
and related compounds on vancomycin-resistant enterococci
(VRE). Biol Pharm Bull.

[B07] KEGG Pathway (2013). Peptidoglycan biosynthesis - *Streptococcus mutans*
UA159.

[B08] Kim MJ, Kim CS, Ha WH (2010). Antimicrobial effects of oleanolic acid against
*Streptococcus mutans* and *Streptococcus
sobrinus* isolated from a Korean population. Int J Oral Biol.

[B09] Kim MJ, Kim CS, Park JY (2011). Antimicrobial effects of ursolic acid against mutans streptococci
isolated from Koreans. Int J Oral Biol.

[B10] Lee M, Hesek D, Suvorov M (2003). A mechanism-based inhibitor targeting the DD-transpeptidase
activity of bacterial penicillin-binding proteins. J Am Chem Soc.

[B11] Liu J (1995). Pharmacology of oleanolic acid and ursolic acid. J Ethnopharmacol.

[B12] Mainardi JL, Fourgeaud M, Hugonnet JE (2005). A novel peptidoglycan cross-linking enzyme for a
beta-lactam-resistant transpeptidation pathway. J Biol Chem.

[B13] Pinho MG, Filipe SR, de Lencastre H (2001). Complementation of the essential peptidoglycan transpeptidase
function of penicillin-binding protein 2 (PBP2) by the drug resistance
protein PBP2A in *Staphylococcus aureus*. J Bacteriol.

